# Contagious Cerebro-spinal Meningitis of the Horse

**Published:** 1898-02

**Authors:** 


### ABSTRACTS FROM FOREIGN JOURNALS.

### GERMAN.

UNDER THE DIRECTION OF J. PRESTON HOSKINS, PH.D.,
PRINCETON, N. J.

CONTAGIOUS CEREBRO-SPINAL MENINGITIS OF THE HORSE.—During the last few years the attention of veterinarians has been called to a contagious cerebro-spinal meningitis of the horse, a disease which appears to have originated during recent years. Drs. Siedamgrotzky and Schlegel have made an extended study of this equine disease in the province of Saxony, and have related their observations and conclusions in *Archiv. fur Wissenschaft u. Prakt. Thierheilkunde*, Bd. xxii., Heft 4 and 5.

The disease was first recognized in 1879, and in nearly every year since that time has claimed its victims. In 1895, 122 horses, scattered through fifty-one localities, perished. The disease appears suddenly, with a severe chill, though often its coming is announced several days previously by a moderate gastro-intestinal catarrh.

Contagious spinal-meningitis begins usually with psychic disturbances. Sometimes it begins with disturbance in the function of the cephalic and cervical muscles, so that the prehension and mastication of food are impossible; deglutition is, however, undisturbed. There is sometimes tonic spasm of the cervical muscles. Frequently an animal seems to be in constant movement, walking slowly in circles either to the right or left. There is frequently a disturbance in the centres of coördination, so that the animals lose their equilibrium, fall to the ground, and often injure themselves. In rare cases they will bite and kick, almost without provocation, so that they can be approached only with great danger.

The subjective symptoms are: a rise of temperature to 105.5° or 106°; an irregular pulse; defecation somewhat increased in frequency; urine high-colored, often containing albumin. The appetite usually remains good.

These symptoms become more pronounced during the first four to eight days, after which time the disease maintains its intensity with slight exacerbations and remissions, and leads, most frequently, by the tenth to the eighteenth day, to death by paralysis.

Convalescence takes place slowly, and requires several weeks. In case an animal survives three or more weeks, death may finally take place by reason of such complications as fracture, necrosis, inanition, or septicæmia. Convalescence may, however, be incomplete, and in no small number of cases there remains a certain amount of psychic depression, so that these animals manifest all the symptoms of hydrocephalus or "dumminess." Furthermore, these animals may present disturbances of equlibrium, weakness of the lumbar muscles, and quite frequently amaurosis.

Out of 780 animals affected with the disease, 595 died—a mortality of 76 to 80 per cent.

Post-mortem examination shows a well-marked serous leptomeningitis. The changes in the organs belonging to the respiratory and alimentary tracts appear of little value in making a diagnosis of the disease.

The authors report that the disease is most prevalent in country districts, and the horses in the cities are seldom affected. No race of the horse is exempt. Those of middle age are especially susceptible, though neither old nor young are immune. The disease is most active in the spring, and seems always to die out with the beginning of summer. Long-continued confinement within the stable and over-feeding, especially with food which has spoiled, predispose to this disease.

The authors first made experiments to determine whether or not this so-called contagious cerebro-spinal meningitis was an infectious disease. They attempted to spread the disease by placing healthy animals with sick ones, and to ascertain the period of incubation. The first results of these experiments seemed to indicate that it was not an infectious disease. After further investigation they were led to the conclusion tha the unknown cause of the disease existed outside of the horse's body as a facultative saprophyte, and, after the manner of a miasm, could infect a predisposed animal when the conditions were opportune.

By the year 1895 the authors had attempted to isolate and cultivate the unknown organism. These attempts were at first negative, because only mixed cultures could be obtained. Finally, a *motile micrococcus* was found immediately after the death of an animal, which germ grew well upon gelatin, agar, potatoes, and other ordinary culture-media. The most favorable temperature for growth is 38° C. No spores are formed; it is both aërobic and anaërobic, liquefies gelatin, and takes a stain when treated by Graham's method. Attempts to transmit the disease to healthy horses by inoculation with brain-substance and with pure cultures have been successful. The micrococcus does not seem to be pathogenic for the ordinary animals used in the laboratory.

In concluding their observations the authors dealt with the therapeutic measures indicated. The most important point lay in prevention, which consists in isolating the diseased animals and disinfecting the stables.—*Centralb. f. Bakt. u. s. w.*, November, 1896.

